# A Model for Protein Sequence Evolution Based on Selective Pressure for Protein Stability: Application to Hemoglobins

**DOI:** 10.4137/ebo.s3120

**Published:** 2009-08-27

**Authors:** Lorraine Marsh

**Affiliations:** Department of Biology, Long Island University, Brooklyn, NY, 11201, USA. Email:lmarsh@liu.edu

**Keywords:** protein stability, negative selection, protein structure, evolutionary model

## Abstract

Negative selection against protein instability is a central influence on evolution of proteins. Protein stability is maintained over evolution despite changes in underlying sequences. An empirical all-site stability-based model of evolution was developed to focus on the selection of residues arising from their contributions to protein stability. In this model, site rates could vary. A structure-based method was used to predict stationary frequencies of hemoglobin residues based on their propensity to promote protein stability at a site. Sites with destabilizing residues were shown to change more rapidly in hemoglobins than sites with stabilizing residues. For diverse proteins the results were consistent with stability-based selection. Maximum likelihood studies with hemoglobins supported the stability-based model over simple Poisson-based methods. These observations are consistent with suggestions that purifying selection to maintain protein structural stability plays a dominant role in protein evolution.

## Introduction

Much protein selection in evolution is centered on a requirement for protein stability.^1^–^3^ In laboratory studies a significant proportion of random substitutions lead to reduced protein stability^4^–^6^ supporting the concept that much selection may act on genes encoding unstable proteins. Selection and mutation rate determine stationary frequencies for amino acids at any given site. For marginally destabilizing residues, the unstable allele would not be eliminated immediately, but the stationary frequency of the allele would be determined by selection.

Evolutionary analyses often use protein structure to inform analyses. Approaches using measures correlated to protein structural stability have been used to study evolutionary relationships and explain patterns of residue change^7^,^8^ Structural information in general (much of which indirectly reflects the need to form a folded protein) has explanatory power and improves understanding of evolutionary processes of substitution.^9^–^12^ The role of protein structure in evolution is still, however, limited in part by our incomplete understanding of requirements for protein folding.

Empirical models for protein evolution rely on external information in their analyses. Substitution matrices are prime examples of precalculated values applied to evolutionary analysis.^13^–^15^ The processed information derived from substitution patterns for a diverse group of proteins are used to provide rate and frequency values that can be applied to specific sites of other proteins.^16^ Though substitution matrices have generally been generated from aligned sequence matrices, force field or mutagenesis data have also been used.^1^,^15^ The biophysical approaches to generation of substitution matrices represent an interesting effort to produce an evolutionary model based on sophisticated analyses of protein structure.

Most substitution matrices are statistically optimized to act on a generic site and a single matrix is applied to all sites of a protein. For applications such as homology searching, it is common to use site-specific profiles. Profiles are 20 × n matrices, where n is protein length, that record stationary frequencies for each amino acid, for each site. Profiles derived from protein sequence alignments, though useful, are often in practice less complete than the equivalent substitution matrices because of the requirement to predict amino acid frequencies for each site of each protein.^17^ One alternative approach to predict site stationary frequencies for proteins is to use information derived from the protein structure.^18^ Stationary frequencies might also be calculated using mutational/biophysical-based approaches^1^,^19^ that permit calculation of the contribution of each residue to protein stability with accuracy.^4^,^20^,^21^ Alternative approaches include decision-tree models and methods relying on phylogeny to predict the effects of mutations.^22^,^23^

In this study an evolutionary model is proposed that is centered on negative selection against unstable proteins. The model is based on stationary frequency profiles that provide an all-site record of relative contribution to protein stability of each potential residue at each site. An empirical evolutionary model is proposed under which sites bearing residues that destabilize the protein change most rapidly. Maximum likelihood analysis of phylogeny with this model and phylogenetic reconstruction tests validated the method. The approach presented provides new opportunities for the study of evolutionary processes.

## Methods

### Sequences and structures

Protein sequences of hemoglobins were obtained from NCBI and edited as necessary. Structures were obtained from the Protein Data Bank. Where a structure file was available the protein sequence corresponding to the structure was extracted and used. The accession numbers for proteins used in this work are shown in Table 1. References in the text to ‘9 hemoglobins’ refer to the 9 hemoglobins of Table 1 for which structures were available.

### Determination of site contributions to stability

The focus of this work was the influence of protein stability on evolution. The first step in assessing the effect of residues on hemoglobin folding was to estimate the stability of all possible singly-substituted hemoglobins. This determination required a method capable of estimating stability of a protein structure. Force field-based methods are capable of generating the required energy value. The all-atom Fold-X force field^21^ was chosen because of its accuracy and efficiency in this particular type of estimation. The Fold-X force field has been specifically parameterized with experimental mutagenesis data to accurately determine stability for substitution events.^20^ In our hands, the standard deviation for estimates of stability energies was about 0.25 kcal/mol as assayed by ability to predict sequences. For stability energy assessment the Fold-X force field was used with settings for explicit waters, a salt concentration of 0.15 M and a temperature of 294 °K. The median Fold-X-assessed energy for 10 independent models of each substitution was taken as the energy value for that residue at that site. This system was reproducible in replicate assays. The Fold-X application required an accurate structural model to assess. The homology modeling program Modeller was used because it is capable of modeling the structure of substituted proteins.^24^ The role of Modeller in this process was to generate candidate structures that approximated the conformation of a hemoglobin with a desired single residue substitution. Modeller8v1^24^ was used in local mode generating 10 models per site. Together, the modeling and force field method permitted assay of stability for hemoglobins substituted with all 20 amino acids at every position.

### Stability energy

Stability energies were calculated. The energy of folding for each possible amino acid residue at each protein site was determined. The difference in the stability energy of proteins with varying residues (*a* or *b*) at a site indicated the difference in propensity of proteins with *a* or *b* to fold. It was convenient to use the residue with the highest stability at a site as an index residue for that site and subtract other stability values from this index value. Sometimes this analysis suggested that the observed residue was not the most favorable residue. Energies for hemoglobins were determined in the absence of the heme ligand. Only stability energies of two histidine residues that complex with heme seemed significantly affected by lack of ligand.

### Derivation of residue stationary frequencies from stability energies

Data was converted from stability energy to evolutionary frequency. In the text an evolutionary model is developed that relates stabilization energy, residue frequencies and rates of residue change. Our model required a stationary frequency profile of probabilities of finding a specific amino acid at a specific position of a protein. Therefore one goal was to be able to convert residue stability energy values to residue frequencies. Stability energies of observed residues were enumerated in 0.25 kcal/mol bins in the range 0–3.0 kcal/mol to create an empirical frequency distribution. To fit an exponential curve to the distribution of ΔΔG, least squares regression was employed. For residue *a,* with a given stability energy, ΔΔG_a_, π_i_(*a*) was the derived, normalized, stationary frequency of *a*. Repeating this procedure for each residue at each site, profiles of stationary frequencies were prepared. The human hemoglobin α stationary frequency profile was used for most evolutionary tests. Profiles derived from 9 hemoglobins were used to test models of selection for protein stability. As a control, mock frequency profiles were prepared for which each site had an identical stationary frequency distribution, with frequencies taken from those of proteins as a whole (“null model”)^13^ or with all residue frequencies identical (all probabilities = 0.05; “unselected model”).

### Comparison of frequency profiles

Profiles of various taxa were compared to determine similarity. Stationary frequency profiles prepared were compared using Jensen-Shannon divergence scoring.^25^–^27^ The divergence score is an information theory-derived method to assess the likelihood that profiles have a common source, such as shared ancestry. The divergence score, D, for stationary frequencies of each site of two profiles, *P* and *Q*, was calculated as D = 0.5∑*_k_* *p_ik_* log_2_(*p_ik_*/(0.5*p_ik_* + 0.5*q_ik_*)) + 0.5∑*_k_* *q_ik_* log_2_ (*q_ik_*/(0.5*p_ik_* + 0.5*q_ik_*)), with *p_ik_* and *q_ik_* the frequencies for a specific site with a specific residue for the two profiles. The final divergence score was averaged over sites. Jensen-Shannon scores can be interpreted as both measure of similarity of selective forces for diverse proteins and as a measure of accuracy in stationary frequency profile estimation since error reduces relatedness. To test comparisons for site specificity a control comparison was made in which hemoglobins were randomized by shifting one of the profiles by one residue relative to the other. To assess significance of Jensen-Shannon scores, profiles were subjected to bootstrap replications, evaluated to produce scores and subjected to the Wilcoxon two-sample test to compare the distribution of profile comparison scores to the distribution of control values.

### Determining stability switches

The evolutionary model required that stabilization states be changeable. To determine the ability of sites to switch from stabilizing to destabilizing and destabilizing to stabilizing, switches were counted on a vertebrate hemoglobin tree. A residue was defined as stabilizing if its stability profile probability was higher than the mean and destabilizing if its stability profile probability was lower than the mean. Sites with movement between higher than average and lower than average frequencies were scored as switching. Conserved sites and other sites whose residue stationary frequencies remained either higher or lower than average for both homologs were scored as not switching. Residues categorized as not switching based on stability state were further categorized based on whether sequence change had occurred at the site. The rate of change at stable and unstable sites was determined. Bootstrap resampling was used to determine significance.

### Model testing with diverse proteins

Most of this study involved hemoglobins. However, it was important to test the models developed on other proteins as well. Proteins of diverse fold type (α, β, α/β) that were small, single domain (or single domains of larger proteins) and not membrane proteins were selected from the Protein Data Bank (PDB). A procedure was developed to test each protein using the evolutionary model of the text. For each site of each protein a random substitution was made. Modeller was used to select sidechain conformations and Fold-X to assess energy. Alternatively the protein was mock substituted then subjected to the same regimen to leave the observed residue intact. ΔΔG was calculated as ΔG_random_−ΔG_observed_. A two-tailed t-test was performed on the ΔΔG results with the null hypothesis that the mean ΔΔG value was 0. To calculate rate ratios, first stationary frequencies were calculated using Eq. 4 of the text. Stationary frequencies were converted to rate ratios by using the relationship π*_i_*(*a*)/π*_i_*(*b*) = rate(*b*)/rate(*a*). All proteins were assumed to be subject to purifying selection. An average rate ratio for random residues/observed residues greater than two for a specific protein was considered evidence that the model had correctly predicted purifying selection for that protein. This analysis also served as a test of protein fitness for the model.

### Maximum likelihood test

In order to test the ability of the model that was developed to function in evolutionary analysis, ML analysis was performed on a hemoglobin tree. Sequences were aligned by ClustalW and edited as necessary. A consistent reference tree was prepared with Mega 3.1 using the N-J method and the Poisson correction setting. Tree internal nodes were reconstructed using PAML 3.14.^28^ The Paml reconstruction used the JTT substitution matrix with a single rate class. In the ML analysis, tree topology and reconstructed ancestral sequences were held constant and branch distances were estimated over the tree. Analysis was performed with the test model and various mock profiles of amino acid stationary frequencies. The test model was used as presented in the text. The AIC statistic (2ln*L*(test)-2ln*L*(control)) was calculated from likelihoods for 100 bootstrap resamplings of sites.

### Phylogenetic reconstruction

Phylogenetic reconstruction was performed to test the consistency of the model developed in the text with other models. Distances between taxa were computed using the model developed in the text (Eq. 2, 3) with stability-based frequency profiles. Sequences were resampled to provide bootstrap replication. Neighbor-Joining (N-J) analysis on the bootstrapped distance matrices was carried out using the Phylip 3.6 program Neighbor.^29^ A consensus tree was created using the Phylip program Consense with the majority rule consensus setting. Non-bootstrapped analyses were carried out with Mega 3.1^30^ and the N-J distance method. Control analyses were carried out with the model developed in the text but with a mock profile with all frequencies in the model set to 0.05. The ratio of observed changes to calculated distance was determined for each pair of sequences using a Poisson distance or alternatively estimated distance and a series of vertebrate hemoglobins.

## Results and Discussion

### An evolutionary model including site contributions to protein stability

A Jukes-Cantor-related model for evolution incorporating protein stability was derived. Much selection in evolution is based on protein functions, but most of it may be based on a simple requirement to maintain stable proteins. The goal of this work was to develop an evolutionary model centered on the requirement for protein stability. Such a model might ultimately be useful for distinguishing sites under selection for function from sites involved only in stability. A three-step process was involved. First an empirical model of evolution, Evolved Stability (ES), based on the relative ability of residues to stabilize a protein was developed. Second, actual protein stability predictions for substituted proteins were generated and used to predict residue amino acid stationary frequencies for each position of the protein. Third, the model was studied to see if the ES model would work with evolutionary problems.

The ES model was developed as a simple empirical evolutionary model that accepted a stationary frequency (stability) profile as input. A profile records the frequency of each residue at each protein site. The model was directed towards the effect of protein stability on rate of residue change and so other aspects of evolution were ignored or treated indirectly. The ES model followed a Jukes-Cantor approach for the initial mutagenesis step, but with a site rate based on residue stationary frequencies. Though intended for use with stationary frequency profiles based on protein stability, the ES model would accept any type of stationary frequency profile. Both forward (first term) and backward (second term) mutations were modeled.

(1) $${\text{dq}}_{i}/\text{dt}=-20\lambda ((\text{q}(1-\pi_{i}(a))-(1-\text{q})(\pi_{i}(a))$$

In the Equation above, q*_i_* represented the probability of a match at site *i* between residue *a* of sequence 1 (ancestral) and residue *b* of sequence 2 (product). The value λ. was the rate of generation of one of the 19 changed amino acid codons and was set to be equal for all amino acid substitutions. The stationary frequency of *a*, π*_i_*(*a*) is the probability of finding residue *a* at site *i*, based on its relative ability to stabilize the protein. In Eq. 1 it is used as a selective value, determining the likelihood that a substitution of *a* to some other residue would be accepted. For substitution to any residue, (1−π*_i_*(*a*))/19 represented the average stationary frequency of a residue that is not residue *a*. So the net result of the model was to introduce substitutions with the probabilities of the stationary frequency profile.

Solving the ordinary differential equation of (Eq. 1) for q*_i_* with the constraint q(0) = 1, and setting distance, d = 19λt yields:

(2) $${\text{q}}_{i}=(1-\pi_{i}(a))\text{exp}(-(20/19)\text{d})+\pi_{i}(a)$$

The predicted probability of site matching at branch length d was q*_i_*. The match frequency q*_i_* could have been calculated as a function of sites in both structures. However it was more compelling to calculate the probability of a specific match in terms of the stationary frequency of the original residue so a single stationary frequency profile was used in the calculation. The frequency of changes was similarly calculated:

(3) $${\text{p}}_{i}=(1-\pi_{i}(a))\;(1-\text{exp}(-(20/19)\text{d}))$$

Using this approach the rate of protein sequence change could be viewed as a property of just the ancestral protein rather than a property of a pair of proteins. Rate of change became a function of ancestral protein site stability under this model with proteins that had an excess of sites promoting unfolding exhibiting a higher rate of change. This perspective has been extended to link mutability directly to the global stability of the protein.^31^ Equations 2 (*a* = *b*) and 3 (*a* ≠ *b*) simplified to a Jukes and Cantor-like model if the residue stationary frequencies were set to be equal in probability. Under those conditions the limiting distribution of amino acid matches would be 0.05 and all residues would contribute equally to protein stability. For the ES model the equilibrium amino acid frequencies at each site are identical to the stationary frequency profile for that site. Though the ES model is specifically targeted towards selection based on protein stability, it would be possible to modify the site stationary probability profile to include other forms of selection. The ES model neglects second site interactions and indel formation. These might be important in some cases.

### Protein stability is a selected feature of hemoglobins

The distribution of site contributions to protein stability was calculated. Using the ES model required information about the relative probability of different residues at a site. This could be calculated from structural information by first calculating the free energy of each substitution and then calculating the stationary frequency of that substitution based on that energy. The protein stability for substituted sites of hemoglobins was predicted using a cycle of *in silico* protein mutagenesis, protein modeling, and protein energy scoring. The relative stability of >2000 possible single substitutions in each of 9 hemoglobins was estimated.

**Figure 1:**
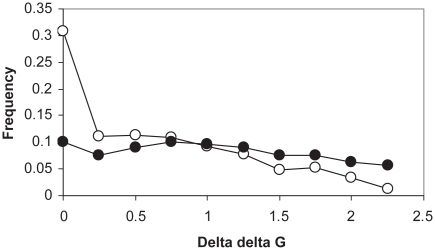
Selection for stability in hemoglobins. Open circles, mean distribution of ΔΔG values of residues observed to occur in 9 hemoglobins (subject to selection); closed circles, mean distribution of ΔΔG values of all possible substitutions, estimated for 9 hemoglobins (unselected). ΔΔG estimated by Modeller-Fold-X analysis of protein structures.

Figure 1 shows the probability distribution of ΔΔG values for two sets of residues in 9 hemoglobins: all potential substitutions, and residues observed to occur in the proteins. The distribution of the stabilities for all possible substitutions (which included both residues that would permit folding and those which would not be compatible with folding) was relatively flat, consistent with the lack of selection for stability. The distribution of stabilities for observed residues on the other hand was roughly exponential with many values skewed toward very stable values. 30.8% of residues were in the most stable state (11.3% would be random) suggesting that stability represents a protein feature subject to selection for these hemoglobins. This result also suggested that error in stability estimation was low.

### Calculation of stationary frequencies for amino acid residues

**Figure 2:**
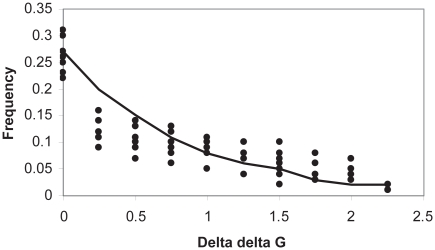
Relationship between stability energy and frequency of observance. Stability is plotted against frequency of occurance. The normalized observed residue stability energy (ΔΔG) distribution for 9 hemoglobins (filled circles) is shown, Also shown is the non-linear least squares fitted exponential curve used to estimate the distribution.

Stability values could be used to produce evolutionary values. The stability values provided information about the energetics of folding. A useful conversion was from the stability for residue *a* at site *i* to the probability, π*_i_* (*a*), of finding residue *a* at that site. The relationship between stability and observed residue probability was roughly exponential. Least squares fitting of data from 9 hemoglobins to an exponential curve yielded the relationship (Fig. 2).

(4) $$\text{Prob}(a)={\text{e}}^{-\Delta \Delta \text{G}(\text{a})*\text{c}}$$

where c was estimated as 1.2, and ΔΔG is the stability value. To calculate stationary frequencies, the expression was normalized to all residues at a site.

(5) $$\pi_{i}(a)={\text{e}}^{-\Delta \Delta \text{G}(a)*\text{c}}/\Sigma_{x=\text{A}\ldots \text{Y}}{\text{e}}^{-\Delta \Delta \text{G}(x)*\text{c}}$$

These normalized values were used to construct hemoglobin stationary frequency profiles (stability profiles) for use in additional analyses. The sum of π*_i_* (*a*) values for all amino acids at a site was 1.0 by definition. An interesting feature of these profiles is that they do not directly incorporate sequence data, only protein structural data. The highest probability residue for a site did not need to be (though it often was) the observed residue.

### Spatial distribution of site contributions to protein stability

**Figure 3:**
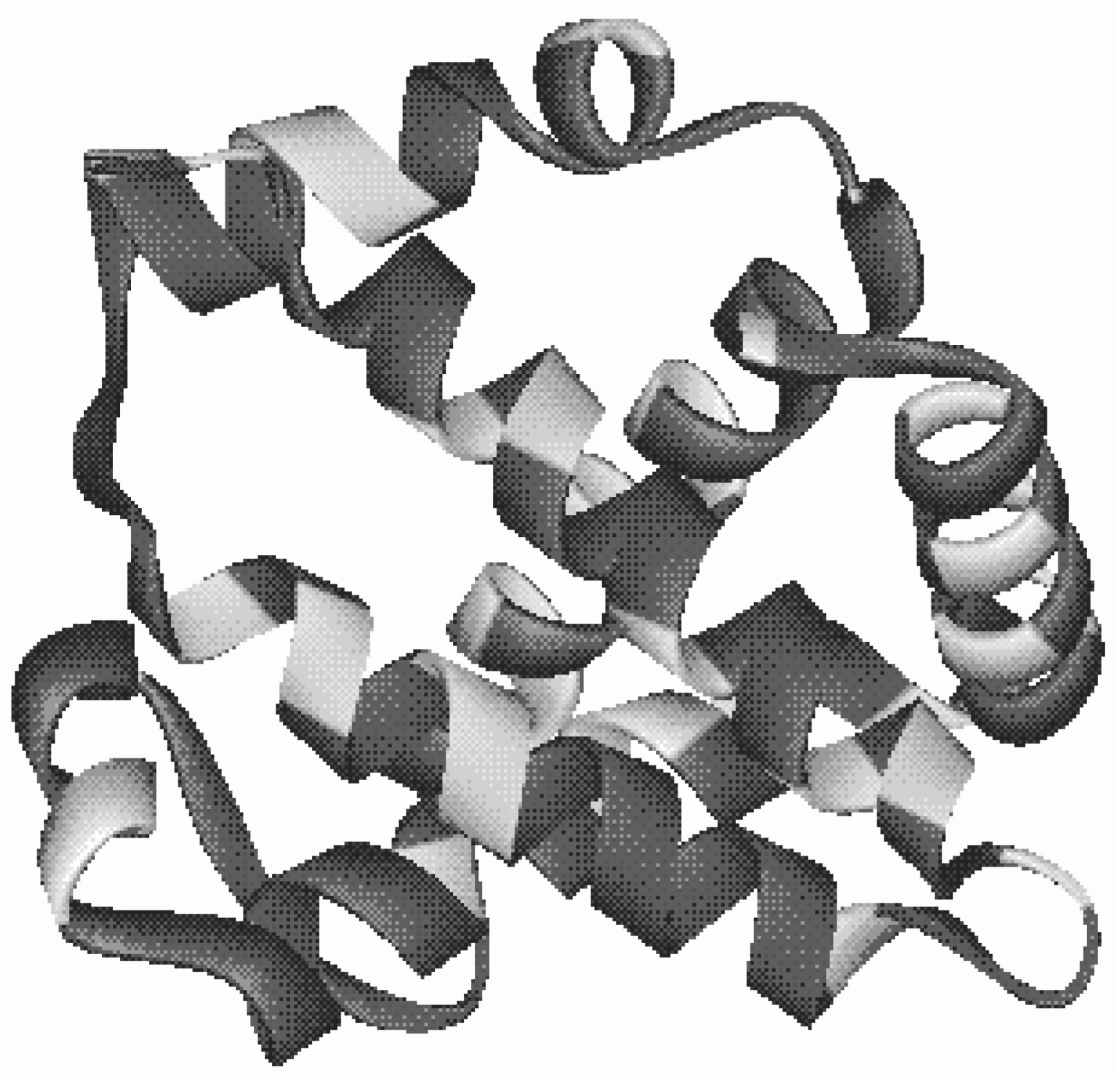
Stability-promoting sites of hemoglobin. The structure of human α hemoglobin is shown with residues predicted to have stationary frequencies greater than the unselected value (0.05) shaded. These sites are predicted to stabilize the protein more than the average site. These sites are potentially under selection and are dispersed throughout helices and loops of the protein.

The spatial distribution of protein stabilization sites was determined for the human α hemoglobin 3-D structure. Ideally, for the ES model of evolution, stabilizing and destabilizing sites should be distributed in most regions of a protein. Stabilizing (high probability) and destabilizing (low probability) residues were analyzed on the folded hemoglobin structure. Stabilizing residues were not clustered but included all secondary elements of hemoglobin (Fig. 3). Stabilizing sites were not concentrated in the core of hemoglobin. This result was consistent with models in which sites involved in protein stability are well dispersed in the protein. Sites may vary in the extent of their roles in folding of the protein, but if a site is ‘stabilizing’ it is promoting stability, even if only incrementally. Stabilizing sites often became destabilizing and vice versa over a hemoglobin lineage (not shown) consistent with the concept that protein stabilization over the course of evolution is an average property of proteins not a fixed property of individual sites.

### Maintenance of stability profiles over evolution

Profiles of different taxa could be compared. The ES model assumed that underlying processes of selection for stability remained unchanged over a lineage. Globins have been shown to retain structural features even as sequence diverges.^32^,^33^ Table 2 shows the divergence scores of hemoglobin stationary frequency profiles from diverse taxa. All of the vertebrate hemoglobin profiles were related and even the clam profile was related to those of human hemoglobins (Table 2) suggesting that the pattern of selection for stability changes very slowly. This conservation also suggests accuracy in profile building since inaccuracy would promote divergence. Hemoglobin structure has been maintained from bacteria to vertebrates^34^ so it is reasonable that stationary frequency profiles would be conserved.^3^,^10^,^35^ Sequences diverged more rapidly, For example, the human α hemoglobin and clam hemoglobin sequences shared only 12.8% sequence identity though their stationary frequency profiles were related. Only the nematode hemoglobin profile failed to show similarity to human hemoglobin profiles. Randomizing site order increased divergence scores, indicating that stationary frequency profiles were site specific. These results suggest that for most purposes any stationary frequency profile on a lineage might be used to predict others. For most of this study, only proteins with more than 30% sequence identity (i.e. vertebrate) were compared. Globin function has been conserved in this lineage. Changes in function in general would not be detected by the ES model. However protein functional changes often involve only a limited portion of the protein. If protein structure is conserved, a significant proportion of residues would still serve primarily to stabilize that structure and the ES model would still be relevant.

### Protein stability affects rate of site change

One significant prediction of the ES model is that sites that stabilize a protein mutate more slowly than sites that tend to destabilize a protein. This concept is a natural consequence of the ES model (Eq. 2, 3). It is also logically intuitive. If a residue at a site has high probability (e.g. a stabilizing residue) then the alternative residues it could change to have lower probability. Therefore change is slower than if the site starts from a low probability state. Since the probability of changing a residue at a site in the ES model is a function solely of its stationary frequency, rate will depend on stabilizing ability. To test this hypothesis on real sequences, vertebrate hemoglobins were compared. A problem in this analysis arises if the ancestral and product residues are not specifiable. To avoid that issue, only residue changes in homologous sites that did not change protein stability values were analyzed. That is, both sites were high stability or both were low stability. For this study a threshold was chosen such that half of sites were in a ‘stabilizing’ pool of sites and half in a ‘destabilizing’ pool. Study of changes over a hemoglobin tree suggested that approximately 54.3% of sites remained either in stabilizing or in destabilizing states and were suitable for this analysis. Table 3 shows that the observed rate of change was higher in the low stability pool of sites than the high stability pool. The low stability sites exhibited a rate of change on average about three times that of the high stability sites. Thus the influence of protein stability on rate is potentially significant in evolutionary terms.

### Generalizability of the ES model

The ES model itself is independent of the method of estimation of ΔΔG. However it was important to determine how well the applied method worked with non-globin proteins. Proteins of a wide variety of folds were tested (Table 4). Membrane proteins and large multi-domain proteins were excluded based on limitations of the assessment method. The difference in energy between unselected (random) amino acid residues and selected (the actually observed) residues was determined. This determination should present a positive ΔΔG if selected residues for sites are, on average, more stabilizing than unselected residues. Table 4 shows that ΔΔG values for tested proteins were positive not neutral (P < 0.001). This result provided evidence that the estimation method was capable of detecting stability differences in diverse protein and provides a simple test of whether the method is appropriate for other proteins. The ΔΔG values would average 0 if the method could not distinguish stabilities. The ΔΔG values were translated into relative evolutionary rates for an average site. The average rate ratio for unselected/selected was 3.55. An unselected amino acid residue for these proteins was predicted to be more likely to change than a selected residue, leading to greater stability for selected residues. Thus this model was consistent with purifying selection, based on stability, for all of the analyzed proteins. The results for α hemoglobin were similar to the results for other proteins. The ES model, at least for these tests, may apply to diverse protein types. These results also suggest an approach to increase efficiency of the ES method. Instead of preparing complete profiles of stationary frequencies for all 20 amino acids, rates of differing residues could be directly compared as above. The profile approach is general, though, and was used for most of this work.

### Maximum likelihood (ML) tests of models

The ES model was compared to other models. A ML analysis of a phylogenetic tree was carried out using the ES model and vertebrate hemoglobins.^36^,^37^ Topology was fixed and likelihood using the ES model (Eq. 4, 5) was maximized over branch lengths (Table 5).^38^ Three conditions were tested by varying the stationary frequency profiles used by the ES model. Residue stationary frequencies were derived from the stability stationary frequency profiles (ES model), or, as a control, set to mock profiles with the average frequencies of amino acids in proteins^13^ or with all amino acid frequencies set to 0.05. The average frequencies of amino acids control reflected selection, but lacked site-specificity (null model). Setting all frequencies to 0.05 (unselected model) eliminated residue selection bias completely. Since these conditions were not nested, likelihoods of ML model pairs were compared using the Akaike Information Criterion (AIC)^39^ (Table 5). The ES model produced higher likelihoods than the null or the unselected models. Bootstrapped AIC comparisons demonstrated that differences were significant and robust. The ML analysis presented here was relatively limited. It would be interesting to see how the ES model performed on a ML or Bayesian analysis where topology and ancestral sequences were inferred.

### Phylogenetic inference

**Figure 4:**
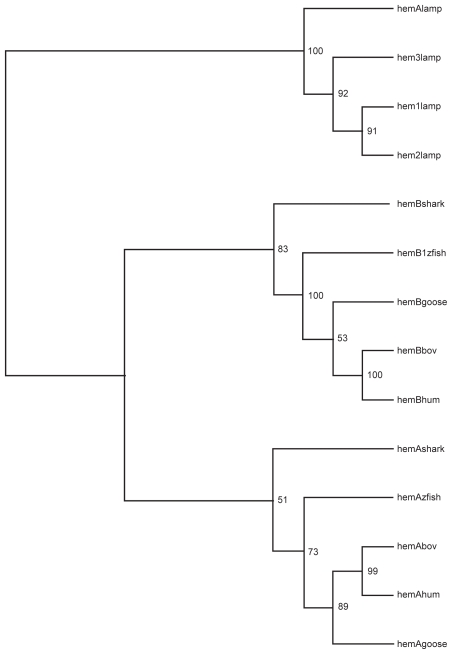
ES-based phylogenetic reconstruction. ES model derived distances based on protein stability predictions were used in Neighbor-Joining phylogenetic infererence of vertebrate hemoglobins. Numbers beside nodes represent support levels from 100 bootstrap replications. Tree was rooted with lamprey hemoglobin. Note that the lamprey, α hemoglobin, β hemoglobin splits are correctly determined.

**Figure 5:**
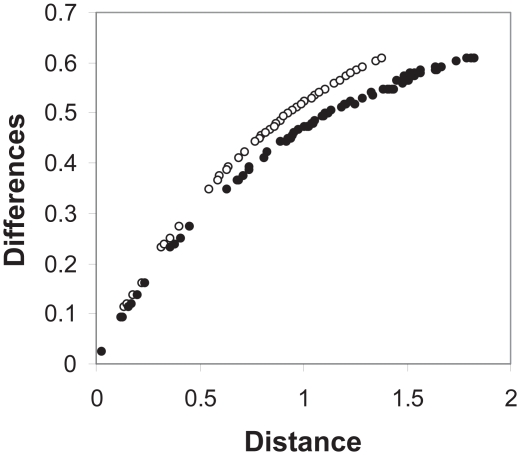
Observed differences vs. distance for metazoan hemoglobins. Sequence differences between hemoglobin proteins (see Fig. 4) were recorded and compared with model predictions of distance (arbitrary units). Unfilled circles, Poisson model; Filled circles, ES model. Distances for a given degree of sequence difference are greater for the ES model.

To test the consistency of the ES model with other models the ES model was applied to phylogenic inference. The model was used to predict distances, which then were used to build a N-J tree (Fig. 4). The ES-generated tree of vertebrate hemoglobins was consistent, with reasonable bootstrap support for nodes. The early split of α and β hemoglobins was supported as well as the grouping of lamprey hemoglobins. The tree topology was similar to that obtained by a Poisson correction of p-values demonstrating consistency of the ES method with existing approaches. Comparison of estimated changes to ES inferred distance (Fig. 5) showed that ES inferred longer distances than the Poisson model. This observation indicated that the ES model better accommodated back mutations over evolution than the Poisson model. There was no reason to believe that the ES model would outperform existing sophisticated methods of phylogenetic inference. The ES model focused on only one (important) aspect of evolution. The observation that the ES model is able to function at all in phylogenetic inference is an indication of the significance of protein stability in evolution. Commonly used methods in phylogenetic inference such as PAM and JTT substitution matrices presumably incorporate stability information, though indirectly.

## Conclusion

A new approach is presented to study the evolution of proteins considering a major selective force: protein stability. The ability to use structural information to predict site contributions to protein stability allowed development of a model of evolution, the ES model, which incorporated stability data in an all-site description of residue change. Including selection for protein stability improved evolutionary predictions for hemoglobins. In the ES model, site contributions to protein stability determine the rate of change. The ES model includes site-specific rates, which most phylogenetic models do not. By explicitly describing the influence of negative selection for protein stability the ES model introduces the possibility of further models that identify selection for other functional aspects of protein evolution. The ES model has some limitations. The method only works for nonmembrane proteins. Structures must exist for proteins under study. Stability measurements were successful on diverse proteins suggesting that the method would be applicable to many protein types. Suitability of a specific protein can be determined by testing. Common methods of protein evolution analysis, e.g. with substitution matrices, capture some of the ES model protein stability component of evolution. However, because they are not all-site rate models some of the specificity of ES may be lost. Stability is an important force in evolution at both the protein and organismal levels. Mutations in hemoglobins that induce unfolding cause serious disease. At the protein level much remains to be understood about how selection for stable proteins acts. The work here provides a framework to study the influence of protein stability on evolution.

## Figures and Tables

**Table 1:** Structures and sequences.

Human α hemoglobin (PDB code: 1A3n_A), hemAhum
Human β hemoglobin (PDB code: 1A3n_B) hemBhum
Bovine α hemoglobin (PDB code: 1hDA_A) hemAbov
Bovine β hemoglobin (PDB code: 1hDA_B) hemBbov
Bar-neck goose α hemoglobin, *Anser indicus* (PDB code: 1C40_A) hemAgoose
Bar-neck goose β hemoglobin, *Anser indicus* (PDB code: 1C40_B) hemBgoose
Sea lamprey monomeric hemoglobin V, *Petromyzon marinus* (PDB code: 1F50_A) hemAlamp
Clam hemoglobin, *Lucina pectinata* (PDB code: 1FLP)
Ascaris hemoglobin, *Ascaris suum* (PDB code: 1Ash_A)
Lamprey hemoglobin 1, *Mordacia mordax* (gLB1_MOrMr) hem1lamp
Lamprey hemoglobin 2, *Mordacia mordax* (gLB2_MOrMr) hem2lamp
Lamprey hemoglobin 3, *Mordacia mordax* (gLB3_MOrMr) hem3lamp
Zebrafish α hemoglobin, *Brachydanio rerio* (HBA_BRARE) hemAzfish
Zebrafish β1 hemoglobin, *Brachydanio rerio* (HB1_BRARE) hemBzfish
Shark hemoglobin α, *Squalus acanthias* (hBA_sQAC) hemAshark
Shark hemoglobin β, *Squalus acanthias* (hBB_sQAC) hemBshark

* Accession codes for protein sequences or PDB codes for structures (which encompass protein sequences).

**Table 2:** Jensen-Shannon Divergence of stationary amino acid probability profiles from diverse hemoglobins.

	Divergence between pairs			
	Human α		Human β	
	Divergence	Control^1^	Divergence	Control
Human α	[0]	0.280	0.076	0.308
Human β	0.076	0.281	[0]	0.297
Bovine α	0.051	0.262	0.071	0.290
Bovine β	0.119	0.264	0.085	0.270
Goose α	0.080	0.260	0.094	0.272
Goose β	0.134	0.308	0.103	0.329
Lamprey	0.109	0.318	0.104	0.325
Clam	0.093	0.291	0.114	0.316
Ascaris	0.230	0.258	0.252	0.270

1 Profile sites randomized to eliminate site-specific correlations. For all pairs except those involving Ascaris there were significant (P < 0.01) differences between test and control by bootstrap tests. A divergence of 0 indicates identity.

**Table 3:** Rate differences between high and low stability sites.

Taxa	Low stability^1^	High stability^2^	Rate ratio^3^
Human α, β	1.550	0.324	4.779**
Cow α, β	1.789	0.333	5.368**
Goose α, β	1.684	0.536	3.144**
Zebrafish α, β	1.174	0.538	2.180**
Human α/Zebrafish α Hemoglobin	0.852	0.268	3.175**

1 Ratio of residues changing to residues not changing at sites ranked as low stability. Only sites where both homologs shared low stability were scored.

2 Ratio of residues changing to residues not changing at sites ranked as high stability.

Only sites where both homologs shared high stability were scored.

3 Ratio of column #1 and column #2.

** (P < 0.01) bootstrap resampling.

**Table 4:** Comparision of stabilization of sites by observed or unselected residues.

PDB Code	Protein	ΔΔG^a^	Rate ratio^b^
1A3N	α-hemoglobin	0.880	2.98
1W6Z	lysozyme	1.224	4.34
1LKK	LCK	0.964	3.18
1LZO	triosephosphate isomerase	1.232	4.39
1FNF	fibronectin(repeat)	1.112	3.80
1KNB	adenovirus fiber protein	0.914	3.00
1WBA	bean albumin	1.039	3.48
1ABR	abrin	1.037	3.47
1ICN	fatty acid binding protein	1.042	3.49
1IHF	integration host factor	0.662	2.21
1RRG	ARF-1	0.975	3.22
1OCT	OCT-1	0.614	2.09
1PUE	ETS	1.018	3.39
1THV	thaumatin	1.463	5.78
2AK3	adenylate kinase	0.940	3.09
1A2P	barnase	1.469	5.88
1X1R	m-RAS	1.101	3.75
1COF	cofilin	1.276	4.63
1FIM	MIF	0.968	3.20
1RCI	ferritin	0.857	2.80
1TTB	transthyretin	0.725	2.39
1ONR	transaldolase B	1.261	4.54
1XIK	ribonucleotide diphosphate reductase	1.166	4.05
1MUP	pheromone binding protein	0.590	2.54
1NHK	nucleoside diphosphate kinase	1.273	4.61
1SVP	sinbis virus capsid	0.725	2.39

a Average site difference in stability between unselected residues and observed residues; kcal/mol. Values were calculated using the Modeller-Foldx method.

b Predicted average ratio at sites of evolutionary rates for unselected residues vs. observed residues. Values were calculated using Eq. 4.

**Table 5:** Comparison of models in ML analysis.

(a) ML with ES model		
*Profile*	ln*L*	
ES-model based	−2489.534	
Null^a^	−2498.238	
Unselected^b^	−2499.096	
(b) Bootstrap test of ES model	
*Compared Profiles*	*Mean AIC*	*P*^c^
ES model-based/null	23.7	<0.001
ES model-based/Unselected	21.7	<0.001

a All sites set to average amino acid frequencies.

b All sites set to frequency of 0.05.

c AIC statistic with 100 bootstrap replicates.
